# Associations between life's essential 8 and the risk of cardiovascular disease in national US population: evidence from NHANES 2005–2018

**DOI:** 10.3389/fcvm.2025.1498240

**Published:** 2025-05-09

**Authors:** Jia Li, Qiaofeng Ge, Yajing Liu, Wenqiang Jia

**Affiliations:** ^1^ Department of Cardiovascular Medicine, Xi’an People’s Hospital (Xi’an Fourth Hospital), Xi’an, Shaanxi, China; ^2^ Department of Traumatic Orthopedics, Xi’an People’s Hospital (Xi’an Fourth Hospital), Xi’an, Shaanxi, China; ^3^ Department of Cardiovascular Medicine, Xi’an No.3 Hospital, Xi’an, Shaanxi, China

**Keywords:** cardiovascular disease, life’s essential 8, cardiovascular health, dose-response, NHANES

## Abstract

**Background:**

Cardiovascular health (CVH) is closely linked to cardiovascular disease (CVD)-specific mortality, yet research on Life's Essential 8 (LE8), a new CVH indicator, and its association with CVD risk is limited.

**Objective:**

This study aims to explore the association between LE8 and CVD risk in US adults.

**Methods:**

A total of 22,298 participants were included in this cross-sectional study from the National Health and Nutrition Examination Survey (NHANES) between 2005 and 2018. LE8 scores were categorized into low, moderate, and high groups. Multivariate logistic regression and restricted cubic spline (RCS) models were employed to examine the association between LE8 scores and CVD risk.

**Results:**

In the multivariate-adjusted model, individuals with moderate and high LE8 scores exhibited a 53% (odds ratio [OR] = 0.47, 95% confidence interval [CI]: 0.41–0.54) and 77% (OR = 0.23, 95% CI: 0.18–0.30) reduction in total CVD risk compared to those with low LE8 scores. RCS analyses revealed an inverse dose-response relationship between LE8 scores and total CVD risk. A consistently negative association was observed between LE8 scores and the risk of CVD subtypes, including congestive heart failure, coronary heart disease, angina pectoris, heart attack, and stroke. Subgroup analyses indicated a more pronounced inverse association between LE8 scores and total CVD risk among participants under 50 years old and with a family history of CVD.

**Conclusions:**

These findings suggest a strong inverse relationship between LE8 and CVD risk. Improving CVH through adherence to LE8 guidelines has significant potential to reduce the burden of CVD.

## Introduction

Cardiovascular disease (CVD) encompasses a range of adverse clinical conditions that impact the cardiovascular system, such as angina pectoris, heart attack, stroke, coronary heart disease, and heart failure (1). The prevalence of CVD is increasing and has emerged as a primary contributor to global mortality and disability (2). In 2020, it is estimated that the worldwide crude prevalence of CVD reached 607.64 million cases, signifying a 29.01% surge from 2010 (2). Concurrently, the global death toll attributed to CVD amounted to 19.05 million, reflecting an 18.71% increase since 2010 (2). Despite notable progress in diagnosing and treating CVD in recent years, the persistently high mortality rates linked to CVD present a grave threat to human life. Consequently, the crucial task of identifying risk factors that can predict CVD risks becomes paramount for facilitating early prevention.

The onset of CVD is intricately connected to a range of risk factors, including genetic, lifestyle, environmental, and social aspects (2). Major contributors to the prevalence of CVD are deemed to be unhealthy lifestyle choices, such as adopting a high-fat diet, insufficient exercise, smoking, and indulging in excessive alcohol consumption (2). Moreover, chronic conditions like hypertension, elevated cholesterol levels, and diabetes mellitus serve as risk factors for CVD, intricately intertwined with the overall health of the cardiovascular system (2). In 2010, the American Heart Association (AHA) launched Life's Simple 7 (LS7) with the goal of improving cardiovascular health (CVH) across the general population through the management of diet, exercise, weight, smoking, blood sugar, blood lipids, and blood pressure (3). LS7 offers comprehensive guidance for preventing CVD, supported by numerous pieces of evidence pointing to a substantial inverse correlation between CVH indicators derived from LS7 and the risk as well as mortality associated with CVD (4–6).

Due to the original definitions of LS7 components not fully capturing inter-individual and intra-individual variations, LS7 may not be entirely suitable for evaluating all health behaviors in the current environment (7). Consequently, in 2022, the AHA further emphasized sleep metrics as a crucial measure of CVH, introducing a new scoring system called Life's Essential 8 (LE8) on the foundation of LS7 to improve CVH in the general population (7). A recent prospective cohort study based on the UK Biobank demonstrated a significant reduction in the risk of coronary heart disease, stroke, and CVD with high LE8 scores (8). Furthermore, a cohort based on the Framingham Heart Study proved that participants with high LE8 scores throughout their lives experienced a significant reduction in the risk of CVD and mortality (9). However, the dose-response relationship between LE8 scores and CVD risk, as well as the association between LE8 scores and different subtypes of CVD remain unclear. Therefore, this cross-sectional study based on the National Health and Nutrition Examination Survey (NHANES) aimed to explore the dose-response relationship between LE8 scores and the risk of CVD in US adults.

## Materials and methods

### Study population

The participants in this study were sourced from the NHANES database, a nationally representative survey conducted by the National Center for Health Statistics (NCHS) under the Centers for Disease Control and Prevention (CDC). NHANES encompasses demographic details, health-related queries, medical examinations, and laboratory assessments. Comprehensive information about NHANES is available on their website (http://www.cdc.gov/nchs/nhanes/index.htm).

For this study, we utilized data from 70,190 subjects who took part in NHANES from 2005 to 2018. Participants were excluded based on the following criteria: (1) age under 20 years (*n* = 30,441), (2) lack of information about the LE8 scores (*n* = 12,763), (3) no available data on the incidence of CVD (*n* = 221), and (4) absence of covariates (*n* = 4,467). Ultimately, this analysis included 22,298 participants (Supplementary Figure S1).

### Life's essential 8 score calculation

The LE8 scores are computed by considering eight components, encompassing four health behaviors (diet quality, physical activity, smoking status, and sleep duration) and four health factors [body mass index (BMI), blood lipids, blood glucose, and blood pressure]. Each LE8 component is assigned a rating on a scale of 0–100. The LE8 scores are determined by calculating the average across these eight components (10, 11). Detailed methods for calculating the LE8 scores using NHANES data are available in Supplementary Table S1.

### Assessment of CVD risk

CVD was determined through self-reported physician diagnoses using a standardized medical condition questionnaire. Participants were asked, “Has a doctor or other health professional ever informed you that you have experienced congestive heart failure, coronary heart disease, angina pectoris, a heart attack, or a stroke?” Participants responding affirmatively to any of these five questions were categorized as having CVD (12).

### Assessment of covariates

Covariates were chosen based on prior research, and standardized questionnaires were utilized to gather information on various factors including age (years), gender (male/female), race/ethnicity (Mexican American, other Hispanic, non-Hispanic white, non-Hispanic black, and other race), family income poverty ratio (≤1.0, 1.1–3.0, >3.0), education levels (less than high school, high school or equivalent, and college or above), marital status (never married, married, and other), and family history of CVD (yes/no). Smoking status was categorized as never smoker (having smoked fewer than 100 cigarettes), former smoker (having smoked at least 100 cigarettes but not currently smoking), and current smoker (currently smoking and having smoked at least 100 cigarettes) (13). Drinking status was classified as non-drinker (individuals who reported consuming less than 12 drinks in their lifetime), low to moderate drinker (≤2 drinks per day for men and ≤1 drink per day for women), or heavy drinker (>2 drinks per day for men and >1 drink per day for women) (14). Leisure-time physical activity was classified as inactive (no leisure-time physical activity), insufficiently active (leisure-time moderate activity 1–5 times per week with metabolic equivalents ranging from 3 to 6 or leisure-time vigorous activity 1–3 times per week with metabolic equivalents >6), and active (those who had more leisure-time moderate or vigorous activity than mentioned above) (15). BMI (kg/m^2^) was calculated as the weight in kilograms divided by the height in meters squared. Hypertension (yes/no) was defined as mean systolic blood pressure ≥140 mmHg and/or mean diastolic blood pressure ≥90 mmHg, self-reported hypertension diagnosed by a professional doctor, or undergoing antihypertensive treatment (16). Diabetes mellitus (yes/no) was defined as a fasting plasma glucose ≥126 mg/dl, 2-h plasma glucose ≥200 mg/dl, hemoglobin A1c ≥ 6.5%, or self-reported diabetes mellitus, diagnosed by a professional doctor, or currently taking insulin/diabetes pills (17). Further details about the questionnaires, examination components, and laboratory procedures can be found in the NHANES reference manuals (18).

### Statistical analysis

Considering the intricate sampling design of NHANES, we incorporated sample weights, clustering, and stratification in our data analysis to produce nationally representative estimates applicable to US residents. Continuous variables were expressed as weighted mean ± standard deviation (SD), while categorical variables were presented as count (weighted percentage). We employed one-way ANOVA and chi-square tests to evaluate differences in continuous and categorical variables among participants in distinct LE8 score groups, respectively.

We conducted logistic regression analyses to estimate the odds ratios (ORs) and 95% confidence intervals (CIs) for CVD risk using three distinct models. Model 1 was not adjusted for covariates, model 2 was adjusted for age, gender, and race/ethnicity, and model 3 was further adjusted for education level, marital status, family income poverty ratio, drinking status, and family history of CVD based on model 2. The LE8 scores were categorized into low (0–49), moderate (50–79), and high (80–100) groups, with the low group serving as the reference. We also explored the associations between LE8 scores and five subtypes of CVD (congestive heart failure, coronary heart disease, angina pectoris, heart attack, and stroke). To delve into the dose-response relationships between LE8 scores and CVD risk, we applied restricted cubic splines (RCS) with three knots placed at the 5th, 50th, and 95th percentiles.

Subgroup analyses were performed to explore the associations between LE8 scores and total CVD risk across various demographic and lifestyle factors, including gender (male, female), age (<50 years, ≥50 years), race/ethnicity (Non-Hispanic White, other races), smoking status (never smoker, former or current smoker), drinking status (non-drinker, drinker), physical activity levels (inactive, insufficiently active or active), hypertension (yes/no), diabetes mellitus (yes/no), family history of CVD (yes/no), and BMI (<25 kg/m^2^, ≥25 kg/m^2^). Additionally, we incorporated the multiplication of variables into the model to assess their interaction with the total CVD risk. This approach may provide additional insights into the complex interplay between LE8 and other determinants of CVD risk. All statistical analyses were conducted using R software (v.4.1.2), and statistical significance was set at a two-sided *P* < 0.05.

## Results

### Baseline characteristics of study participants

The baseline characteristics of the study population are presented in Table 1. Individuals with higher LE8 scores were more likely to be younger, female, of other race, unmarried, had a high education level, and exhibit a higher family income poverty ratio (all *P* < 0.001). Furthermore, participants in the highest LE8 score group showed higher proportions of non-smokers, non-drinkers, and physically active individuals, along with lower proportions of diabetes mellitus, hypertension, and family history of CVD, when compared to those in the lowest group (all *P* < 0.001).

**Table 1:** Characteristics of participants by three categories of LE8 score.

Characteristics	LE8 score				*P* value
	Total	Low (0–49)	Moderate (50–79)	High (80–100)	
No. of participants	22,298	2,830	15,077	4,391	
Age, mean (SD), year	53.35 ± 17.16	59.33 ± 13.50	54.28 ± 16.89	43.58 ± 7.56	<0.001
BMI, mean (SD), kg/m^2^	29.34 ± 6.75	34.09 ± 7.76	29.36 ± 6.27	24.69 ± 3.90	<0.001
Gender, *n* (%)					<0.001
Female	10,980 (51.82)	1,377 (52.52)	7,832 (54.06)	1,771 (41.48)	
Male	11,318 (48.18)	1,453 (47.48)	7,245 (45.94)	2,620 (58.52)	
Race/ethnicity, *n* (%)					<0.001
Mexican American	3,295 (22.46)	379 (20.69)	2,314 (23.51)	602 (19.61)	
Other Hispanic	1,981 (13.34)	231 (12.58)	1,356 (13.58)	394 (13.01)	
Non-Hispanic White	10,499 (26.00)	1,267 (25.79)	7,067 (26.07)	2,165 (25.92)	
Non-Hispanic Black	4,498 (27.39)	818 (36.55)	3,148 (27.65)	532 (17.49)	
Other race	2,025 (10.82)	135 (4.40)	1,192 (9.19)	698 (23.97)	
Education level, *n* (%)					<0.001
Less than high school	4,760 (29.31)	956 (40.16)	3,326 (29.99)	478 (15.99)	
High school or equivalent	5,124 (22.15)	799 (25.56)	3,732 (23.30)	593 (13.95)	
College or above	12,414 (48.54)	1,075 (34.27)	8,019 (46.72)	3,320 (70.05)	
Marital status, *n* (%)					<0.001
Never married	3,893 (15.40)	355 (11.05)	2,407 (14.02)	1,131 (25.56)	
Married	11,812 (52.24)	1,342 (48.38)	8,070 (52.96)	2,400 (52.84)	
Other	6,593 (32.36)	1,133 (40.57)	4,600 (33.02)	860 (21.60)	
Family income poverty ratio, *n* (%)					<0.001
≤1.0	4,270 (23.34)	802 (30.72)	2,829 (22.87)	639 (18.29)	
1.1–3.0	9,226 (45.10)	1,351 (48.58)	6,448 (46.32)	1,427 (36.50)	
>3.0	8,802 (31.56)	677 (20.69)	5,800 (30.80)	2,325 (45.22)	
Smoking status, *n* (%)					<0.001
Never smoker	12,210 (53.81)	757 (28.19)	7,846 (52.67)	3,607 (83.29)	
Former smoker	5,648 (27.04)	827 (32.26)	4,119 (28.68)	702 (14.99)	
Current smoker	4,440 (19.14)	1,246 (39.55)	3,112 (18.65)	82 (1.72)	
Drinking status, *n* (%)					<0.001
Non-drinker	2,665 (14.03)	315 (11.97)	1,741 (13.86)	609 (16.75)	
Low to moderate drinker	11,664 (54.22)	1,582 (56.94)	7,866 (54.37)	2,216 (50.97)	
Heavy drinker	7,969 (31.75)	933 (31.09)	5,470 (31.77)	1,566 (32.28)	
Leisure-time physical activity, *n* (%)					<0.001
Inactive	10,822 (53.84)	2,292 (82.58)	7,626 (54.18)	904 (24.85)	
Insufficiently active	8,548 (34.40)	461 (14.70)	5,762 (34.81)	2,325 (51.52)	
Active	2,928 (11.76)	77 (2.72)	1,689 (11.01)	1,162 (23.63)	
Hypertension, *n* (%)					<0.001
Yes	9,512 (49.64)	2,095 (77.88)	6,798 (51.01)	619 (16.65)	
No	12,786 (50.36)	735 (22.12)	8,279 (48.99)	3,772 (83.35)	
Diabetes mellitus, *n* (%)					<0.001
Yes	3,722 (22.29)	1,227 (48.83)	2,392 (20.89)	103 (2.88)	
No	18,576 (77.71)	1,603 (51.17)	12,685 (79.11)	4,288 (97.12)	
Family history of CVD, *n* (%)					<0.001
Yes	2,880 (12.17)	554 (17.09)	1,972 (12.08)	354 (7.83)	
No	19,418 (87.83)	2,276 (82.91)	13,105 (87.92)	4,037 (92.17)	
LE8 score, mean (SD)	64.84 ± 14.35	42.21 ± 6.20	64.92 ± 8.13	86.18 ± 4.86	<0.001
HEI-2015 diet score, mean (SD)	41.26 ± 31.35	23.93 ± 25.46	40.45 ± 30.41	61.34 ± 29.36	<0.001
Leisure-time physical activity score, mean (SD)	64.45 ± 44.32	25.09 ± 39.75	66.47 ± 43.34	93.44 ± 18.99	<0.001
Nicotine exposure score, mean (SD)	71.21 ± 38.41	48.26 ± 42.66	71.24 ± 37.97	93.09 ± 17.53	<0.001
Sleep health score, mean (SD)	79.86 ± 26.30	64.04 ± 31.32	80.75 ± 25.23	91.16 ± 16.79	<0.001
BMI score, mean (SD)	58.81 ± 33.38	35.22 ± 30.40	58.16 ± 32.07	84.26 ± 21.98	<0.001
Blood lipid score, mean (SD)	63.22 ± 30.13	46.67 ± 29.57	62.44 ± 29.10	82.44 ± 23.85	<0.001
Blood glucose score, mean (SD)	77.74 ± 28.27	54.22 ± 29.69	78.66 ± 26.98	96.28 ± 12.36	<0.001
Blood pressure score, mean (SD)	62.20 ± 32.68	40.26 ± 30.02	61.23 ± 31.55	87.45 ± 20.92	<0.001

BMI, body mass index; CVD, cardiovascular disease; HEI, healthy eating index; LE8, life's essential 8; OR, odds ratio; SD, standard deviation. Continuous variables are presented as weighted means ± SD and categorical variables are presented as *n* (weighted percentage).

### Association between life's essential 8 scores and total CVD risk

**Figure 1:**
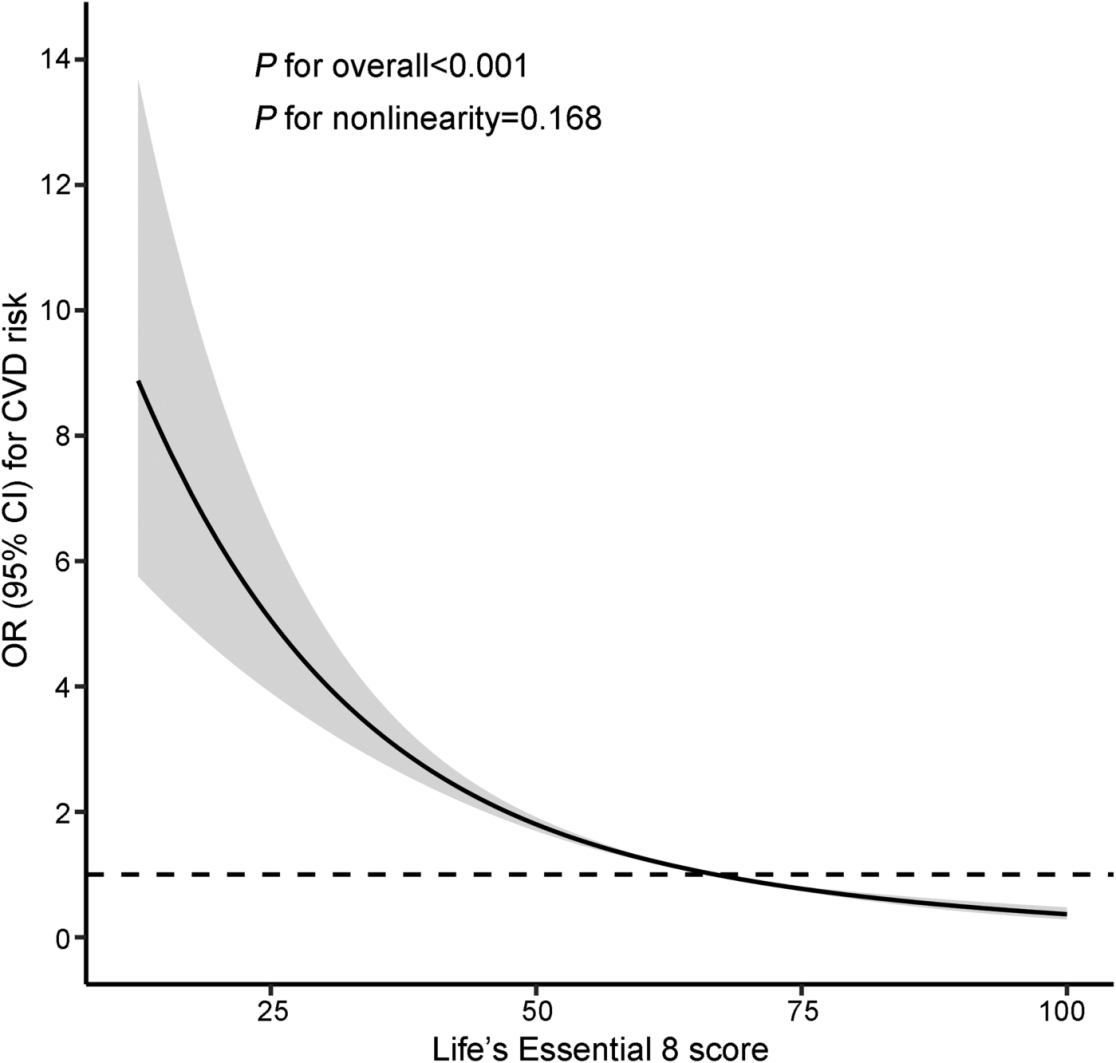
Restricted cubic splines of LE8 scores and the risk of CVD. The model was adjusted for age, gender, race/ethnicity, education level, marital status, family income poverty ratio, drinking status, and family history of CVD. Knots were placed at the 5th, 50th, and 95th percentiles. The reference value was set at the 50th percentile. CI, confidence interval; CVD, cardiovascular disease; LE8, Life's Essential 8; OR, odds ratio.

Table 2 displays the relationship between LE8 scores and total CVD risk. In the multivariate-adjusted model, participants in the moderate LE8 score group (50–79) experienced a 53% reduction in total CVD risk (OR = 0.47, 95% CI: 0.41–0.54), and those in the highest LE8 score group (80–100) showed a 77% reduction (OR = 0.23, 95% CI: 0.18–0.30) in total CVD risk (*P* for trend < 0.05), compared to the lowest group. In the continuous analyses, the OR for the risk of total CVD was 0.71 (95% CI: 0.68–0.74) per 10-point increase in LE8 scores after adjusting for potential covariates. This negative association was still observed in the RCS analysis (*P* for overall <0.05), showing a monotonically decreasing dose-response relationship between LE8 scores and total CVD risk (*P* for nonlinearity = 0.168; Figure 1).

**Table 2:** Associations of LE8 score with CVD risk.

Variables	LE8 score			P for trend	Per 10-point increase
	Low (0–49)	Moderate (50–79)	High (80–100)		
	OR (95%CI)	OR (95%CI)	OR (95%CI)		
P for trend					
Model 1	1.00 (Reference)	0.41 (0.36–0.46)	0.11 (0.09–0.14)	<0.001	0.64 (0.62–0.67)
Model 2	1.00 (Reference)	0.44 (0.38–0.51)	0.20 (0.15–0.26)	<0.001	0.69 (0.66–0.72)
Model 3	1.00 (Reference)	0.47 (0.41–0.54)	0.23 (0.18–0.30)	<0.001	0.71 (0.68–0.74)
HEI-2015 diet score					
Model 1	1.00 (Reference)	0.95 (0.84–1.07)	0.88 (0.76–1.01)	0.081	0.98 (0.96–1.00)
Model 2	1.00 (Reference)	0.79 (0.69–0.90)	0.64 (0.55–0.75)	<0.001	0.94 (0.92–0.95)
Model 3	1.00 (Reference)	0.83 (0.72–0.95)	0.69 (0.59–0.81)	<0.001	0.95 (0.93–0.97)
Leisure-time physical activity score					
Model 1	1.00 (Reference)	0.49 (0.37–0.67)	0.51 (0.44–0.58)	<0.001	0.93 (0.91–0.94)
Model 2	1.00 (Reference)	0.63 (0.46–0.86)	0.66 (0.57–0.76)	<0.001	0.95 (0.94–0.97)
Model 3	1.00 (Reference)	0.68 (0.49–0.93)	0.68 (0.59–0.79)	<0.001	0.96 (0.94–0.97)
Nicotine exposure score					
Model 1	1.00 (Reference)	1.49 (1.29–1.74)	0.62 (0.53–0.72)	<0.001	0.96 (0.95–0.98)
Model 2	1.00 (Reference)	0.77 (0.65–0.91)	0.57 (0.48–0.68)	<0.001	0.94 (0.93–0.96)
Model 3	1.00 (Reference)	0.82 (0.69–0.97)	0.62 (0.52–0.74)	<0.001	0.95 (0.94–0.97)
Sleep health score					
Model 1	1.00 (Reference)	0.67 (0.56–0.80)	0.63 (0.55–0.71)	<0.001	0.93 (0.91–0.95)
Model 2	1.00 (Reference)	0.71 (0.59–0.86)	0.60 (0.52–0.68)	<0.001	0.92 (0.90–0.94)
Model 3	1.00 (Reference)	0.76 (0.63–0.91)	0.65 (0.56–0.75)	<0.001	0.93 (0.91–0.95)
BMI score					
Model 1	1.00 (Reference)	0.79 (0.70–0.90)	0.64 (0.56–0.74)	<0.001	0.95 (0.93–0.96)
Model 2	1.00 (Reference)	0.65 (0.57–0.75)	0.60 (0.51–0.69)	<0.001	0.93 (0.91–0.94)
Model 3	1.00 (Reference)	0.67 (0.58–0.77)	0.60 (0.52–0.71)	<0.001	0.93 (0.91–0.95)
Blood lipid score					
Model 1	1.00 (Reference)	0.52 (0.43–0.64)	1.50 (1.33–1.70)	<0.001	1.01 (0.99–1.03)
Model 2	1.00 (Reference)	0.64 (0.52–0.78)	1.52 (1.34–1.73)	<0.001	1.03 (1.01–1.05)
Model 3	1.00 (Reference)	0.64 (0.52–0.79)	1.56 (1.37–1.78)	<0.001	1.03 (1.01–1.05)
Blood glucose score					
Model 1	1.00 (Reference)	0.40 (0.34–0.46)	0.21 (0.18–0.24)	<0.001	0.82 (0.80–0.83)
Model 2	1.00 (Reference)	0.39 (0.34–0.46)	0.32 (0.27–0.37)	<0.001	0.86 (0.84–0.88)
Model 3	1.00 (Reference)	0.41 (0.35–0.48)	0.33 (0.29–0.39)	<0.001	0.87 (0.85–0.89)
Blood pressure score					
Model 1	1.00 (Reference)	0.35 (0.30–0.41)	0.36 (0.32–0.41)	<0.001	0.84 (0.82–0.85)
Model 2	1.00 (Reference)	0.55 (0.46–0.64)	0.85 (0.74–0.98)	0.051	0.94 (0.92–0.96)
Model 3	1.00 (Reference)	0.56 (0.48–0.67)	0.86 (0.75–0.99)	0.068	0.94 (0.92–0.96)

BMI, body mass index; CI, confidence interval; CVD, cardiovascular disease; HEI, healthy eating index; LE8, life's essential 8; OR, odds ratio.

Model 1: unadjusted.

Model 2: adjusted for age, gender, and race/ethnicity.

Model 3: adjusted for age, gender, race/ethnicity, education level, marital status, family income poverty ratio, drinking status, and family history of CVD.

Likewise, for the individual components of LE8 scores, we observed that higher scores in healthy eating index (HEI)-2015 diet, physical activity, nicotine exposure, sleep duration, BMI, blood glucose, and blood pressure were significantly associated with a reduced risk of total CVD. However, concerning blood lipid scores, we found a 36% reduction in the risk of total CVD (OR = 0.64, 95% CI: 0.52–0.79) in the moderate group and a 56% increase (OR = 1.56, 95% CI: 1.37–1.78) in the highest group, compared to the lowest group.

### Association between life's essential 8 scores and subtypes of CVD risk

Supplementary Tables S2–S6 present the results regarding the associations between LE8 scores and various subtypes of CVD risk. Higher LE8 scores demonstrated a consistent negative association with the risk of congestive heart failure, coronary heart disease, angina pectoris, heart attack, and stroke (all *P* for trend <0.05). With each 10-point increase in LE8 scores, the ORs (95%CIs) for the risk of congestive heart failure, coronary heart disease, angina pectoris, heart attack, and stroke were 0.66 (0.61–0.71), 0.79 (0.74–0.84), 0.74 (0.68–0.80), 0.69 (0.64–0.74), and 0.72 (0.67–0.77), respectively.

### Subgroup analyses

The subgroup analyses of the association between LE8 scores and total CVD risk are outlined in Table 3. Our findings indicated that the inverse association between LE8 scores and total CVD risk appears to be more pronounced in the <50 years age group compared to the ≥50 years age group (*P* for interaction < 0.001). Additionally, a noteworthy interactive effect was observed concerning a family history of CVD, revealing a stronger inverse association among participants with a family history of CVD (OR = 0.14, 95% CI: 0.08–0.26) than those without a family history of CVD (OR = 0.25, 95% CI: 0.19–0.33) in the highest group (*P* for interaction = 0.037). Furthermore, we examined the relationship between LE8 scores and total CVD risk based on other factors, and the results indicated that the inverse associations were generally consistent across subgroups stratified by gender, race/ethnicity, smoking status, drinking status, leisure-time physical activity, diabetes mellitus, hypertension, and BMI (all *P* for interaction >0.05).

**Table 3:** Stratified analyses of the associations between LE8 score and CVD risk.

Characteristic	LE8 score			P for trend	Per 10-point increase	P for interaction
	Low (0–49)	Moderate (50–79)	High (80–100)			
Sex						
Male	1.00 (Reference)	0.48 (0.40–0.58)	0.26 (0.18–0.36)	<0.001	0.72 (0.67–0.77)	0.366
Female	1.00 (Reference)	0.46 (0.37–0.58)	0.18 (0.12–0.28)	<0.001	0.69 (0.64–0.74)	
Age						
<50	1.00 (Reference)	0.29 (0.22–0.39)	0.10 (0.05–0.18)	<0.001	0.59 (0.54–0.65)	<0.001
≥50	1.00 (Reference)	0.52 (0.45–0.60)	0.27 (0.20–0.36)	<0.001	0.74 (0.70–0.77)	
Smoking status						
Never smoker	1.00 (Reference)	0.44 (0.33–0.58)	0.19 (0.13–0.27)	<0.001	0.69 (0.64–0.74)	0.110
Former or current smoker	1.00 (Reference)	0.52 (0.44–0.62)	0.37 (0.26–0.53)	<0.001	0.74 (0.70–0.78)	
Drinking status						
Non-drinker	1.00 (Reference)	0.49 (0.41–0.57)	0.25 (0.19–0.32)	<0.001	0.72 (0.68–0.76)	0.713
Drinker	1.00 (Reference)	0.49 (0.41–0.57)	0.25 (0.19–0.32)	<0.001	0.72 (0.68–0.76)	
Leisure-time physical activity						
Inactive	1.00 (Reference)	0.50 (0.42–0.60)	0.21 (0.13–0.34)	<0.001	0.70 (0.66–0.75)	0.541
Insufficiently active or active	1.00 (Reference)	0.42 (0.30–0.59)	0.24 (0.15–0.37)	<0.001	0.73 (0.67–0.79)	
Hypertension						
Yes	1.00 (Reference)	0.57 (0.49–0.66)	0.35 (0.25–0.50)	<0.001	0.78 (0.74–0.82)	0.279
No	1.00 (Reference)	0.41 (0.27–0.62)	0.26 (0.15–0.44)	<0.001	0.69 (0.62–0.77)	
Diabetes mellitus						
Yes	1.00 (Reference)	0.56 (0.45–0.69)	0.24 (0.10–0.58)	<0.001	0.76 (0.69–0.83)	0.701
No	1.00 (Reference)	0.55 (0.44–0.68)	0.33 (0.25–0.45)	<0.001	0.76 (0.72–0.81)	
Family history of CVD						
Yes	1.00 (Reference)	0.55 (0.43–0.71)	0.14 (0.08–0.26)	<0.001	0.70 (0.65–0.77)	0.037
No	1.00 (Reference)	0.46 (0.39–0.54)	0.25 (0.19–0.33)	<0.001	0.71 (0.68–0.75)	
BMI						
<25	1.00 (Reference)	0.42 (0.27–0.67)	0.20 (0.11–0.34)	<0.001	0.65 (0.58–0.73)	0.810
≥25	1.00 (Reference)	0.49 (0.42–0.57)	0.25 (0.18–0.36)	<0.001	0.72 (0.68–0.77)	

BMI, body mass index; CI, confidence interval; CVD, cardiovascular disease; LE8, life's essential 8; OR, odds ratio.

Model was adjusted for age, gender, race/ethnicity, education level, marital status, family income poverty ratio, drinking status, and family history of CVD.

## Discussion

In this extensive cross-sectional study of US adults, we found that individuals with higher LE8 scores exhibit a reduced risk of total CVD. Moreover, there was a significant linear dose-response relationship between LE8 scores and total CVD risk. For the individual LE8 components, the results showed significant negative associations between the scores of HEI-2015 diet, physical activity, nicotine exposure, sleep duration, BMI, blood glucose, and blood pressure and the risk of total CVD. This association was consistent across different subtypes of CVD. Our findings underscore the significant potential of LE8 as a novel CVH indicator in predicting future CVD risk.

Consistent with previous studies, we identified that adults with higher LE8 scores had reduced CVD risk. In a recent meta-analysis (4), Radovanovic and colleagues examined the association between CVH metrics, as measured by LS7, and the risk of CVD events. This study involved approximately 6.5 million participants, and the findings indicated that having a higher number of ideal CVH metrics significantly reduces the risk of developing composite CVD. Moreover, consistent with our findings, they also discovered a more robust negative association between LS7 scores and the risk of coronary heart disease, myocardial infarction, and stroke (4). Recently, Li and colleagues investigated the relationship between the LE8 scores and the occurrence of CVD in a prospective study that involved 137,794 participants without prior CVD and discovered that higher LE8 scores were significantly associated with a reduced risk of coronary heart disease, stroke, and CVD (8).

For the individual health behaviors, we found that the incidence of CVD was reduced by 38% for participants with high nicotine exposure scores compared to those with low nicotine exposure scores. The utilization of both combustible cigarettes and e-cigarettes presents a significant public health issue in the US (19, 20). Increasing evidence from cohort studies and meta-analyses indicates a link between smoking and increased risk of CVD (21–24). Our results verified this association by taking into account the updated definition of LE8 by the AHA, which now encompasses nicotine exposure from both inhaled nicotine delivery systems and exposure to secondhand smoke. In the current study, we found that sleep health was an influencing factor in CVD risk. Accumulating studies have indicated that both short (<7 h) and long (>8 h) sleep durations are associated with an increased risk of CVD events and mortality (25–27). We also observed that a higher HEI-2015 diet score was linked to a lower risk of CVD in the present study. A noteworthy modification in LE8 involves the assessment of diet. While LS7 suggested evaluating diet scores based on just five components—intake of fruits and vegetables, fish, fiber-rich whole grains, sodium, and sugar-sweetened beverages—LE8 introduces a more comprehensive approach (3, 28). The AHA now recommends a population-level assessment using the DASH dietary pattern or HEI-2015, and an individual assessment utilizing the Mediterranean dietary pattern (28). In 2020, Hu et al. investigated 12,413 participants from the Atherosclerosis Risk in Communities (ARIC) study and revealed that higher HEI-2015 scores were associated with a reduced risk of CVD events among US adults (29). It is important to highlight our discovery that blood glucose, BMI, and blood pressure emerged as the primary individual contributors to CVD risk among US adults, aligning with findings from earlier studies (30–33). However, an opposite result was found in our study, with the moderate group of blood lipid scores being associated with a reduced risk of CVD and the highest group of blood lipid scores being associated with an increased risk of CVD. Although previous studies have shown that low-density lipoprotein cholesterol (LDL-C) is a well-recognized risk factor for CVD and high-density lipoprotein cholesterol (HDL-C) is a protective factor for CVD, some recent studies have suggested that there may be a nonlinear association between LDL-C and HDL-C and CVD risk (34, 35). This result calls for a reassessment of the conventional understanding of the role of blood lipids in CVH. Additional investigations are necessary to elucidate the underlying mechanisms and potential confounding factors contributing to this unexpected association. Notably, among the individual LE8 metrics, blood glucose emerged as a key factor in reducing CVD risk in our study. This finding underscores the critical role of glycemic control in cardiovascular health and aligns with prior evidence linking diabetes management to reduced CVD risk. Further research is needed to explore the relative contributions of individual LE8 metrics in diverse populations.

Furthermore, our stratified analysis revealed a robust negative association between the LE8 scores and the risk of CVD in younger adults, suggesting that early intervention to improve CVH may be more effective in reducing CVD risk. Over the last two decades, a significant prevalence of CVD risk factors, including obesity, physical inactivity, and poor diet, has been noted among young individuals residing in developed countries (36). The Coronary Artery Risk Development in Young Adults (CARDIA) study investigated whether embracing a healthy lifestyle during young adulthood correlates with having a low CVD risk profile in middle age and suggest that maintaining a healthy lifestyle during young adulthood is closely linked to possessing a low CVD risk profile in middle age (37). Therefore, improving CVH in younger adults holds great significance in reducing the CVD burden in the US.

Additionally, we observed a substantial negative association between the LE8 scores and CVD risk among participants with a family history of CVD. Numerous studies have clarified the strong link between a family history of CVD and the risk of CVD events (38–40). A prospective study from the Framingham Heart Study revealed that parental CVD independently predicted future events in middle-aged US adults (39). Hence, timely interventions to improve CVH in individuals with a family history of CVD could result in greater benefits.

To our knowledge, our study is the first to explore the association and the dose-response relationship between CVH metrics measured by LE8 and CVD risk in US adults based on a large, nationally representative sample. However, there are some limitations that need to be acknowledged. Firstly, the assessment of behavioral factors relies primarily on questionnaires, introducing the possibility of information bias and misclassification. Secondly, when investigating the relationship between LE8 scores and CVD risk, although we adjusted for several confounders, the observed results may still be affected by potential confounding factors. Thirdly, the majority of study participants belonged to the white ethnicity, highlighting the necessity for further exploration among other racial and ethnic groups. Moreover, our study lacked a comparison across different geographic regions. CVH metrics, such as LE8, may vary significantly across regions due to differences in socioeconomic status, healthcare access, cultural practices, and environmental factors. Future research should aim to investigate geographic variations in LE8 scores and their associations with CVD risk to provide more region-specific insights and recommendations. Lastly, in our current study, we were unable to track participants' LE8 scores at different times, preventing us from elucidating the impact of potential lifestyle changes on the development of CVD.

## Conclusions

In conclusion, this comprehensive cross-sectional study revealed a strong inverse relationship between LE8 scores as a new indicator of CVH and the risk of various cardiovascular conditions, encompassing total CVD, coronary heart disease, congestive heart failure, angina pectoris, heart attack, and stroke among US adults. Furthermore, a distinct dose-response relationship was observed between the LE8 scores and the risk of CVD. These results emphasize the crucial need to reduce the risk of CVD by implementing the LE8 guidelines.

## Data Availability

Publicly available datasets were analyzed in this study. This data can be found here: https://wwwn.cdc.gov/nchs/nhanes/Default.aspx.
