# Supplementary material for: Associations between life's essential 8 and the risk of cardiovascular disease in national US population: evidence from NHANES 2005–2018
Source: Front Cardiovasc Med. 2025 May 9;12:1498240. doi: 10.3389/fcvm.2025.1498240 (PMC12098637; doi:10.3389/fcvm.2025.1498240)
Supplement: Supplementary file 1 [file Datasheet1.pdf]

Supplementary Materials

Associations between Life's Essential 8 and the risk of cardiovascular disease in national US population: evidence from NHANES 2005–2018

Jia Li[1], Qiaofeng Ge[2], Yajing Liu[3], Wenqiang Jia[1,#]

[1] Department of Cardiovascular Medicine, Xi'an People's Hospital (Xi'an Fourth Hospital), Xi’an, Shaanxi, China;

[2] Department of Traumatic Orthopedics, Xi'an People's Hospital (Xi'an Fourth Hospital), Xi’an, Shaanxi, China;

[3] Department of Cardiovascular Medicine, Xi’an No.3 Hospital, Xi’an, Shaanxi, China.

[#]Corresponding author:

Wenqiang Jia, Department of Cardiovascular Medicine, Xi'an People's Hospital (Xi'an Fourth Hospital), Xi’an, Shaanxi, China.

E-mail: drjiawenqiang@163.com

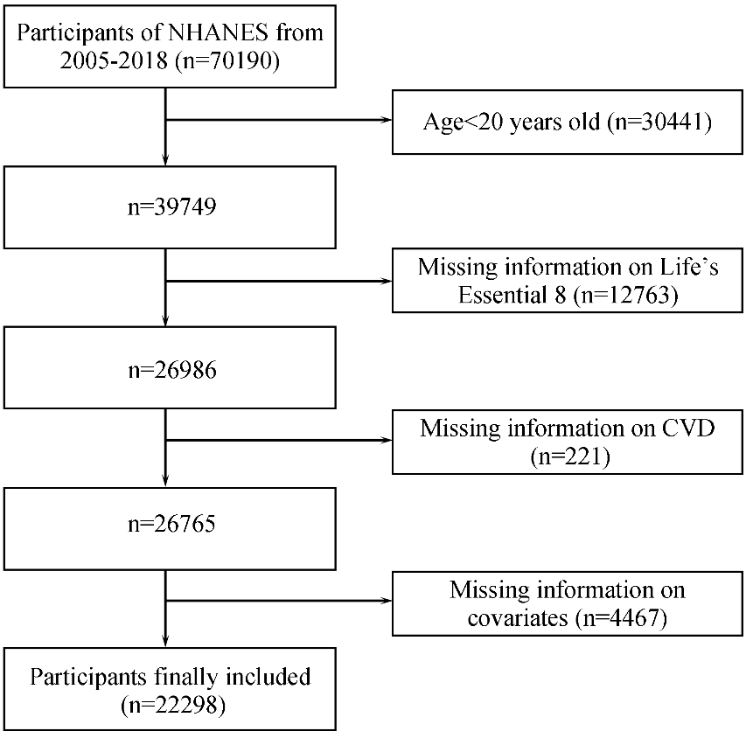

Supplementary Fig. 1 The flowchart for the selection of the study participants.

Supplementary Table 1. Definition and scoring approach for the LE8 score

Domain	Components	Measurement	Scoring of CVH Metric	
			**Metric**	**Points**
Health Behaviors	Diet	HEI-2015 diet score percentile	≥ 95th percentile (top/ideal diet)	100
			75th-94th percentile	80
			50th-74th percentile	50
			25th-49th percentile	25
			1st-24th percentile (bottom/least ideal quartile)	0
			≥150 minutes	100
	Leisure-time physical activity	Self-reported minutes of moderate or vigorous physical activity per week	120–149 minutes	90
			90–119 minutes	80
			60–89 minutes	60
			30–59 minutes	40
			1–29 minutes	20
			0 minutes	0
	Nicotine exposure[a]	Self-reported use of cigarettes or inhaled nicotine-delivery system	Never smoker	100
			Former smoker, quit≥5y	75
			Former smoker, quit 1-<5y	50
			Former smoker, quit <1 y, or currently using inhaled NDS	25
			Current smoker	0
	Sleep health	Self-reported average hours of sleep per night	7-<9	100
			9-<10	90
			6-<7	70
			5-<6 or ≥10	40
			4-<5	20
			<4	0
Health Factors	BMI	Body weight (kg) divided by height squared (m^2)	<25.0	100

		25.0-29.9	70
		30.0-34.9	30
		35.0-39.9	15
		≥ 40.0	0
Blood lipids[b]	Plasma total and HDL-cholesterol with calculation of non-HDLcholesterol (mg/dL)	<130	100
		130-159	60
		160-189	40
		190-219	20
		≥220	0
Blood glucose	FBG (mg/dL) or casual HbA1c (%)	No history of diabetes and FBG <100 (or HbA1c <5.7)	100
		No diabetes and FBG 100-125 (or HbA1c 5.7-6.4) (Pre-diabetes)	60
		Diabetes with HbA1c <7.0	40
		Diabetes with HbA1c 7.0-7.9	30
		Diabetes with HbA1c 8.0-8.9	20
		Diabetes with Hb A1c 9.0-9.9	10
		Diabetes with HbA1c ≥10.0	0
Blood pressure[c]	Appropriately measured systolic and diastolic blood pressure (mmHg)	<120/<80 (Optimal)	100
		120-129/<80 (Elevated)	75
		130-139 or 80-89 (Stage I HTN)	50
		140-159 or 90-99	25
		≥160 or ≥100	0

Abbreviations: BMI, body mass index; CVH, cardiovascular health; FBG, fasting blood glucose; HEI, healthy eating index; HbA1c, Hemoglobin A1c; HDL, high-density lipoprotein; LE8, Life's Essential 8.

[a] Subtract 20 points (unless score is 0) for living with active indoor smoker in home.

[b] If drug-treated level, subtract 20 points.

[c] Subtract 20 points if treated level.

Supplementary Table 2. Associations of LE8 score with congestive heart failure risk

	LE8 score				
	**Low (0–49) OR (95%CI)**	**Moderate (50–79) OR (95%CI)**	**High (80–100) OR (95%CI)**	***P* for trend**	**Per 10-point increase**
LE8 score					
Model 1	1.00 (Reference)	0.35 (0.29-0.43)	0.06 (0.04-0.10)	<0.001	0.61 (0.57-0.64)
Model 2	1.00 (Reference)	0.40 (0.32-0.49)	0.11 (0.06-0.19)	<0.001	0.64 (0.60-0.69)
Model 3	1.00 (Reference)	0.42 (0.34-0.52)	0.13 (0.07-0.22)	<0.001	0.66 (0.61-0.71)
HEI-2015 diet score					
Model 1	1.00 (Reference)	1.15 (0.92-1.44)	0.85 (0.68-1.06)	0.44	0.98 (0.95-1.01)
Model 2	1.00 (Reference)	1.02 (0.81-1.28)	0.67 (0.53-0.85)	0.005	0.95 (0.92-0.97)
Model 3	1.00 (Reference)	1.07 (0.85-1.36)	0.73 (0.57-0.93)	0.051	0.96 (0.93-0.99)
Leisure-time physical activity score					
Model 1	1.00 (Reference)	0.42 (0.23-0.76)	0.43 (0.35-0.52)	<0.001	0.91 (0.89-0.93)
Model 2	1.00 (Reference)	0.54 (0.29-1.00)	0.54 (0.44-0.67)	<0.001	0.93 (0.91-0.96)
Model 3	1.00 (Reference)	0.59 (0.32-1.09)	0.56 (0.45-0.70)	<0.001	0.94 (0.92-0.96)
Nicotine exposure score					
Model 1	1.00 (Reference)	1.61 (1.23-2.10)	0.69 (0.54-0.89)	0.017	0.97 (0.95-0.99)
Model 2	1.00 (Reference)	0.91 (0.69-1.19)	0.69 (0.52-0.92)	0.015	0.96 (0.93-0.98)
Model 3	1.00 (Reference)	1.00 (0.76-1.33)	0.77 (0.58-1.03)	0.117	0.97 (0.94-1.00)
Sleep health score					
Model 1	1.00 (Reference)	0.64 (0.48-0.85)	0.65 (0.53-0.81)	0.001	0.93 (0.90-0.96)
Model 2	1.00 (Reference)	0.70 (0.52-0.94)	0.67 (0.53-0.84)	0.002	0.93 (0.90-0.97)
Model 3	1.00 (Reference)	0.74 (0.55-0.99)	0.74 (0.59-0.93)	0.023	0.95 (0.91-0.98)
BMI score					
Model 1	1.00 (Reference)	0.58 (0.48-0.71)	0.48 (0.37-0.62)	<0.001	0.90 (0.88-0.93)
Model 2	1.00 (Reference)	0.49 (0.40-0.59)	0.45 (0.34-0.59)	<0.001	0.88 (0.85-0.91)

Model 3	1.00 (Reference)	0.50 (0.41-0.61)	0.45 (0.35-0.60)	<0.001	0.88 (0.85-0.91)
Blood lipid score					
Model 1	1.00 (Reference)	0.42 (0.29-0.60)	1.41 (1.11-1.79)	<0.001	1.01 (0.97-1.04)
Model 2	1.00 (Reference)	0.51 (0.35-0.73)	1.33 (1.04-1.69)	0.003	1.01 (0.98-1.05)
Model 3	1.00 (Reference)	0.51 (0.35-0.74)	1.34 (1.06-1.71)	0.002	1.02 (0.98-1.06)
Blood glucose score					
Model 1	1.00 (Reference)	0.31 (0.25-0.38)	0.16 (0.13-0.19)	<0.001	0.79 (0.77-0.82)
Model 2	1.00 (Reference)	0.31 (0.25-0.39)	0.24 (0.20-0.30)	<0.001	0.82 (0.80-0.85)
Model 3	1.00 (Reference)	0.32 (0.26-0.41)	0.26 (0.21-0.32)	<0.001	0.83 (0.80-0.86)
Blood pressure score					
Model 1	1.00 (Reference)	0.32 (0.24-0.42)	0.45 (0.36-0.55)	<0.001	0.85 (0.83-0.87)
Model 2	1.00 (Reference)	0.49 (0.37-0.66)	1.01 (0.80-1.27)	0.865	0.95 (0.92-0.98)
Model 3	1.00 (Reference)	0.52 (0.39-0.69)	1.01 (0.80-1.28)	0.853	0.95 (0.92-0.99)

Abbreviations: BMI, body mass index; CI, confidence interval; CVD, cardiovascular disease; HEI, healthy eating index; LE8, life’s essential 8; OR, odds ratio.

Model 1: unadjusted.

Model 2: adjusted for age, gender, and race/ethnicity.

Model 3: adjusted for age, gender, race/ethnicity, education level, marital status, family income poverty ratio, drinking status, and family history of CVD.

Supplementary Table 3. Associations of LE8 score with coronary heart disease risk

	LE8 score				
	Low (0–49) OR (95%CI)	Moderate (50–79) OR (95%CI)	High (80–100) OR (95%CI)	*P* for trend	Per 10-point increase
LE8 score					
Model 1	1.00 (Reference)	0.53 (0.44-0.63)	0.20 (0.14-0.28)	<0.001	0.74 (0.70-0.78)
Model 2	1.00 (Reference)	0.55 (0.45-0.66)	0.33 (0.23-0.47)	<0.001	0.78 (0.74-0.83)
Model 3	1.00 (Reference)	0.56 (0.46-0.68)	0.34 (0.24-0.50)	<0.001	0.79 (0.74-0.84)
HEI-2015 diet score					
Model 1	1.00 (Reference)	1.09 (0.89-1.33)	1.14 (0.96-1.36)	0.108	1.01 (0.99-1.04)
Model 2	1.00 (Reference)	0.94 (0.76-1.16)	0.88 (0.73-1.06)	0.16	0.98 (0.95-1.00)
Model 3	1.00 (Reference)	0.96 (0.77-1.20)	0.91 (0.75-1.10)	0.329	0.98 (0.96-1.01)
Leisure-time physical activity score					
Model 1	1.00 (Reference)	0.47 (0.29-0.77)	0.64 (0.54-0.77)	<0.001	0.95 (0.93-0.97)
Model 2	1.00 (Reference)	0.60 (0.35-1.02)	0.84 (0.69-1.02)	0.08	0.98 (0.96-1.00)
Model 3	1.00 (Reference)	0.65 (0.39-1.1)	0.85 (0.70-1.04)	0.115	0.98 (0.96-1.00)
Nicotine exposure score					
Model 1	1.00 (Reference)	1.80 (1.43-2.27)	0.73 (0.58-0.93)	0.037	0.98 (0.96-1.00)
Model 2	1.00 (Reference)	0.84 (0.66-1.07)	0.69 (0.54-0.89)	0.006	0.96 (0.94-0.99)
Model 3	1.00 (Reference)	0.85 (0.66-1.08)	0.70 (0.53-0.91)	0.011	0.96 (0.94-0.99)
Sleep health score					
Model 1	1.00 (Reference)	0.93 (0.73-1.19)	0.98 (0.79-1.21)	0.965	1.00 (0.97-1.03)
Model 2	1.00 (Reference)	1.01 (0.79-1.29)	0.90 (0.73-1.11)	0.25	0.98 (0.95-1.01)
Model 3	1.00 (Reference)	1.03 (0.80-1.33)	0.96 (0.77-1.19)	0.609	0.99 (0.96-1.02)
BMI score					
Model 1	1.00 (Reference)	0.92 (0.76-1.11)	0.71 (0.57-0.89)	0.003	0.97 (0.95-0.99)
Model 2	1.00 (Reference)	0.69 (0.56-0.84)	0.61 (0.48-0.77)	<0.001	0.94 (0.91-0.96)

Model 3	1.00 (Reference)	0.71 (0.58-0.87)	0.63 (0.50-0.80)	<0.001	0.94 (0.91-0.97)
Blood lipid score					
Model 1	1.00 (Reference)	0.41 (0.29-0.58)	1.83 (1.54-2.17)	<0.001	1.02 (0.99-1.04)
Model 2	1.00 (Reference)	0.50 (0.35-0.72)	1.77 (1.48-2.12)	<0.001	1.03 (1.00-1.06)
Model 3	1.00 (Reference)	0.51 (0.35-0.72)	1.77 (1.48-2.12)	<0.001	1.04 (1.00-1.07)
Blood glucose score					
Model 1	1.00 (Reference)	0.37 (0.30-0.45)	0.21 (0.17-0.26)	<0.001	0.83 (0.81-0.85)
Model 2	1.00 (Reference)	0.36 (0.29-0.44)	0.31 (0.25-0.40)	<0.001	0.86 (0.83-0.89)
Model 3	1.00 (Reference)	0.37 (0.29-0.46)	0.33 (0.26-0.41)	<0.001	0.87 (0.84-0.89)
Blood pressure score					
Model 1	1.00 (Reference)	0.38 (0.30-0.47)	0.40 (0.34-0.48)	<0.001	0.85 (0.83-0.87)
Model 2	1.00 (Reference)	0.57 (0.45-0.72)	0.89 (0.73-1.09)	0.328	0.94 (0.92-0.97)
Model 3	1.00 (Reference)	0.59 (0.46-0.74)	0.88 (0.72-1.07)	0.25	0.94 (0.92-0.97)

Abbreviations: BMI, body mass index; CI, confidence interval; CVD, cardiovascular disease; HEI, healthy eating index; LE8, life’s essential 8; OR, odds ratio.

Model 1: unadjusted.

Model 2: adjusted for age, gender, and race/ethnicity.

Model 3: adjusted for age, gender, race/ethnicity, education level, marital status, family income poverty ratio, drinking status, and family history of CVD.

Supplementary Table 4. Associations of LE8 score with angina pectoris risk

	LE8 score				
	**Low (0–49)**<br>**OR (95%CI)**	**Moderate (50–79)**<br>**OR (95%CI)**	**High (80–100)**<br>**OR (95%CI)**	***P* for trend**	**Per 10-point increase**
LE8 score					
Model 1	1.00 (Reference)	0.37 (0.29-0.48)	0.15 (0.09-0.23)	<0.001	0.69 (0.64-0.74)
Model 2	1.00 (Reference)	0.40 (0.31-0.51)	0.23 (0.14-0.37)	<0.001	0.72 (0.67-0.78)
Model 3	1.00 (Reference)	0.41 (0.32-0.53)	0.25 (0.15-0.42)	<0.001	0.74 (0.68-0.80)
HEI-2015 diet score					
Model 1	1.00 (Reference)	0.84 (0.64-1.11)	0.96 (0.75-1.24)	0.545	0.99 (0.96-1.03)
Model 2	1.00 (Reference)	0.73 (0.55-0.96)	0.74 (0.57-0.97)	0.014	0.96 (0.92-0.99)
Model 3	1.00 (Reference)	0.76 (0.57-1.01)	0.79 (0.60-1.04)	0.053	0.97 (0.93-1.00)
Leisure-time physical activity score					
Model 1	1.00 (Reference)	0.65 (0.37-1.16)	0.66 (0.54-0.80)	<0.001	0.95 (0.93-0.97)
Model 2	1.00 (Reference)	0.81 (0.45-1.46)	0.84 (0.68-1.05)	0.127	0.98 (0.96-1.00)
Model 3	1.00 (Reference)	0.87 (0.48-1.59)	0.87 (0.69-1.09)	0.224	0.98 (0.96-1.00)
Nicotine exposure score					
Model 1	1.00 (Reference)	1.57 (1.20-2.05)	0.75 (0.56-1.01)	0.101	0.98 (0.95-1.00)
Model 2	1.00 (Reference)	0.87 (0.66-1.15)	0.69 (0.50-0.95)	0.025	0.96 (0.93-0.99)
Model 3	1.00 (Reference)	0.89 (0.68-1.17)	0.73 (0.52-1.01)	0.066	0.96 (0.93-0.99)
Sleep health score					
Model 1	1.00 (Reference)	0.68 (0.49-0.94)	0.69 (0.54-0.89)	0.019	0.95 (0.91-0.98)
Model 2	1.00 (Reference)	0.72 (0.52-1.00)	0.65 (0.50-0.83)	0.002	0.93 (0.90-0.97)
Model 3	1.00 (Reference)	0.75 (0.53-1.05)	0.69 (0.53-0.89)	0.01	0.94 (0.91-0.98)
BMI score					
Model 1	1.00 (Reference)	0.80 (0.63-1.01)	0.38 (0.27-0.53)	<0.001	0.92 (0.89-0.95)
Model 2	1.00 (Reference)	0.66 (0.53-0.83)	0.34 (0.24-0.49)	<0.001	0.89 (0.86-0.92)

Model 3	1.00 (Reference)	0.69 (0.55-0.87)	0.36 (0.26-0.51)	<0.001	0.90 (0.87-0.93)
Blood lipid score					
Model 1	1.00 (Reference)	0.46 (0.32-0.67)	1.44 (1.18-1.76)	<0.001	1.00 (0.98-1.03)
Model 2	1.00 (Reference)	0.57 (0.39-0.82)	1.46 (1.19-1.80)	<0.001	1.02 (0.99-1.06)
Model 3	1.00 (Reference)	0.58 (0.40-0.84)	1.48 (1.20-1.83)	<0.001	1.03 (0.99-1.06)
Blood glucose score					
Model 1	1.00 (Reference)	0.43 (0.33-0.57)	0.21 (0.17-0.27)	<0.001	0.82 (0.80-0.85)
Model 2	1.00 (Reference)	0.43 (0.33-0.57)	0.30 (0.23-0.39)	<0.001	0.85 (0.83-0.88)
Model 3	1.00 (Reference)	0.45 (0.34-0.60)	0.32 (0.25-0.41)	<0.001	0.86 (0.83-0.89)
Blood pressure score					
Model 1	1.00 (Reference)	0.39 (0.29-0.52)	0.47 (0.37-0.60)	<0.001	0.87 (0.84-0.89)
Model 2	1.00 (Reference)	0.56 (0.42-0.75)	0.91 (0.70-1.19)	0.634	0.94 (0.91-0.98)
Model 3	1.00 (Reference)	0.59 (0.44-0.79)	0.91 (0.70-1.18)	0.595	0.95 (0.91-0.98)

Abbreviations: BMI, body mass index; CI, confidence interval; CVD, cardiovascular disease; HEI, healthy eating index; LE8, life’s essential 8; OR, odds ratio.

Model 1: unadjusted.

Model 2: adjusted for age, gender, and race/ethnicity.

Model 3: adjusted for age, gender, race/ethnicity, education level, marital status, family income poverty ratio, drinking status, and family history of CVD.

Supplementary Table 5. Associations of LE8 score with heart attack risk

	LE8 score				
	Low (0–49)<br>OR (95%CI)	Moderate (50–79)<br>OR (95%CI)	High (80–100)<br>OR (95%CI)	*P* for trend	Per 10-point increase
LE8 score					
Model 1	1.00 (Reference)	0.39 (0.32-0.47)	0.11 (0.08-0.16)	<0.001	0.64 (0.60-0.68)
Model 2	1.00 (Reference)	0.42 (0.34-0.51)	0.20 (0.13-0.29)	<0.001	0.66 (0.62-0.71)
Model 3	1.00 (Reference)	0.45 (0.36-0.56)	0.23 (0.16-0.35)	<0.001	0.69 (0.64-0.74)
HEI-2015 diet score					
Model 1	1.00 (Reference)	0.86 (0.71-1.04)	0.83 (0.67-1.03)	0.057	0.97 (0.94-0.99)
Model 2	1.00 (Reference)	0.74 (0.61-0.89)	0.66 (0.53-0.81)	<0.001	0.93 (0.91-0.96)
Model 3	1.00 (Reference)	0.78 (0.64-0.95)	0.72 (0.59-0.90)	0.001	0.95 (0.92-0.97)
Leisure-time physical activity score					
Model 1	1.00 (Reference)	0.47 (0.28-0.80)	0.50 (0.41-0.60)	<0.001	0.93 (0.91-0.94)
Model 2	1.00 (Reference)	0.60 (0.35-1.04)	0.61 (0.50-0.75)	<0.001	0.95 (0.93-0.97)
Model 3	1.00 (Reference)	0.67 (0.39-1.15)	0.64 (0.52-0.79)	<0.001	0.95 (0.93-0.97)
Nicotine exposure score					
Model 1	1.00 (Reference)	1.38 (1.10-1.73)	0.50 (0.38-0.65)	<0.001	0.95 (0.92-0.97)
Model 2	1.00 (Reference)	0.70 (0.55-0.89)	0.49 (0.38-0.65)	<0.001	0.93 (0.9-0.95)
Model 3	1.00 (Reference)	0.76 (0.60-0.97)	0.56 (0.43-0.74)	<0.001	0.94 (0.91-0.97)
Sleep health score					
Model 1	1.00 (Reference)	0.61 (0.47-0.78)	0.62 (0.50-0.76)	<0.001	0.93 (0.90-0.96)
Model 2	1.00 (Reference)	0.64 (0.50-0.83)	0.58 (0.47-0.73)	<0.001	0.92 (0.89-0.95)
Model 3	1.00 (Reference)	0.69 (0.53-0.88)	0.65 (0.52-0.81)	<0.001	0.93 (0.90-0.96)
BMI score					
Model 1	1.00 (Reference)	0.71 (0.59-0.84)	0.63 (0.50-0.79)	<0.001	0.95 (0.92-0.97)
Model 2	1.00 (Reference)	0.55 (0.45-0.67)	0.57 (0.45-0.72)	<0.001	0.92 (0.89-0.95)

Model 3	1.00 (Reference)	0.57 (0.46-0.70)	0.58 (0.45-0.73)	<0.001	0.92 (0.89-0.95)
Blood lipid score					
Model 1	1.00 (Reference)	0.44 (0.31-0.62)	1.56 (1.29-1.89)	<0.001	1.02 (0.99-1.04)
Model 2	1.00 (Reference)	0.53 (0.37-0.75)	1.49 (1.23-1.81)	<0.001	1.03 (1.00-1.06)
Model 3	1.00 (Reference)	0.53 (0.37-0.76)	1.52 (1.25-1.84)	<0.001	1.03 (1.00-1.06)
Blood glucose score					
Model 1	1.00 (Reference)	0.47 (0.38-0.58)	0.21 (0.17-0.26)	<0.001	0.82 (0.80-0.85)
Model 2	1.00 (Reference)	0.48 (0.38-0.61)	0.33 (0.26-0.42)	<0.001	0.86 (0.83-0.89)
Model 3	1.00 (Reference)	0.51 (0.41-0.65)	0.35 (0.28-0.45)	<0.001	0.87 (0.84-0.90)
Blood pressure score					
Model 1	1.00 (Reference)	0.35 (0.28-0.44)	0.43 (0.36-0.52)	<0.001	0.85 (0.83-0.87)
Model 2	1.00 (Reference)	0.53 (0.41-0.67)	0.95 (0.78-1.16)	0.74	0.95 (0.92-0.98)
Model 3	1.00 (Reference)	0.55 (0.43-0.70)	0.95 (0.78-1.16)	0.728	0.95 (0.93-0.98)

Abbreviations: BMI, body mass index; CI, confidence interval; CVD, cardiovascular disease; HEI, healthy eating index; LE8, life's essential 8; OR, odds ratio.

Model 1: unadjusted.

Model 2: adjusted for age, gender, and race/ethnicity.

Model 3: adjusted for age, gender, race/ethnicity, education level, marital status, family income poverty ratio, drinking status, and family history of CVD.

Supplementary Table 6. Associations of LE8 score with stroke risk

	LE8 score				
	**Low (0–49) OR (95%CI)**	**Moderate (50–79) OR (95%CI)**	**High (80–100) OR (95%CI)**	***P* for trend**	**Per 10-point increase**
LE8 score					
Model 1	1.00 (Reference)	0.41 (0.34-0.49)	0.08 (0.05-0.14)	<0.001	0.64 (0.60-0.67)
Model 2	1.00 (Reference)	0.47 (0.39-0.58)	0.15 (0.09-0.26)	<0.001	0.69 (0.64-0.74)
Model 3	1.00 (Reference)	0.51 (0.42-0.63)	0.18 (0.11-0.32)	<0.001	0.72 (0.67-0.77)
HEI-2015 diet score					
Model 1	1.00 (Reference)	0.91 (0.75-1.11)	0.67 (0.52-0.87)	0.004	0.96 (0.93-0.99)
Model 2	1.00 (Reference)	0.78 (0.63-0.95)	0.50 (0.39-0.65)	<0.001	0.92 (0.89-0.95)
Model 3	1.00 (Reference)	0.81 (0.66-1.00)	0.55 (0.42-0.71)	<0.001	0.93 (0.90-0.96)
Leisure-time physical activity score					
Model 1	1.00 (Reference)	0.41 (0.25-0.67)	0.42 (0.34-0.52)	<0.001	0.91 (0.89-0.93)
Model 2	1.00 (Reference)	0.53 (0.33-0.86)	0.57 (0.46-0.71)	<0.001	0.94 (0.92-0.96)
Model 3	1.00 (Reference)	0.58 (0.36-0.94)	0.60 (0.49-0.75)	<0.001	0.95 (0.93-0.97)
Nicotine exposure score					
Model 1	1.00 (Reference)	1.33 (1.05-1.68)	0.55 (0.44-0.69)	<0.001	0.96 (0.94-0.98)
Model 2	1.00 (Reference)	0.75 (0.59-0.97)	0.49 (0.39-0.62)	<0.001	0.94 (0.91-0.96)
Model 3	1.00 (Reference)	0.81 (0.62-1.05)	0.54 (0.42-0.69)	<0.001	0.95 (0.92-0.97)
Sleep health score					
Model 1	1.00 (Reference)	0.63 (0.50-0.81)	0.62 (0.51-0.75)	<0.001	0.92 (0.90-0.95)
Model 2	1.00 (Reference)	0.70 (0.54-0.91)	0.64 (0.52-0.78)	<0.001	0.93 (0.90-0.95)
Model 3	1.00 (Reference)	0.74 (0.57-0.97)	0.70 (0.57-0.86)	0.002	0.94 (0.91-0.97)
BMI score					
Model 1	1.00 (Reference)	0.83 (0.68-1.01)	0.77 (0.62-0.95)	0.011	0.97 (0.95-0.99)
Model 2	1.00 (Reference)	0.75 (0.61-0.92)	0.77 (0.62-0.97)	0.012	0.96 (0.94-0.99)

Model 3	1.00 (Reference)	0.78 (0.63-0.96)	0.79 (0.62-0.99)	0.022	0.97 (0.94-0.99)
Blood lipid score					
Model 1	1.00 (Reference)	0.57 (0.43-0.74)	1.29 (1.06-1.56)	<0.001	0.99 (0.97-1.02)
Model 2	1.00 (Reference)	0.69 (0.52-0.91)	1.24 (1.02-1.51)	0.006	1.00 (0.97-1.04)
Model 3	1.00 (Reference)	0.70 (0.53-0.92)	1.25 (1.02-1.54)	0.006	1.01 (0.98-1.04)
Blood glucose score					
Model 1	1.00 (Reference)	0.44 (0.34-0.55)	0.26 (0.21-0.31)	<0.001	0.84 (0.82-0.87)
Model 2	1.00 (Reference)	0.46 (0.36-0.59)	0.42 (0.34-0.52)	<0.001	0.89 (0.86-0.92)
Model 3	1.00 (Reference)	0.49 (0.38-0.63)	0.45 (0.36-0.56)	<0.001	0.90 (0.87-0.93)
Blood pressure score					
Model 1	1.00 (Reference)	0.39 (0.31-0.49)	0.38 (0.32-0.46)	<0.001	0.85 (0.82-0.87)
Model 2	1.00 (Reference)	0.61 (0.48-0.78)	0.86 (0.70-1.07)	0.199	0.94 (0.91-0.97)
Model 3	1.00 (Reference)	0.63 (0.49-0.81)	0.87 (0.70-1.08)	0.248	0.95 (0.92-0.98)

Abbreviations: BMI, body mass index; CI, confidence interval; CVD, cardiovascular disease; HEI, healthy eating index; LE8, life’s essential 8; OR, odds ratio.

Model 1: unadjusted.

Model 2: adjusted for age, gender, and race/ethnicity.

Model 3: adjusted for age, gender, race/ethnicity, education level, marital status, family income poverty ratio, drinking status, and family history of CVD.
